# Heart Rate Variability Synchronizes When Non-experts Vocalize Together

**DOI:** 10.3389/fphys.2020.00762

**Published:** 2020-09-08

**Authors:** Sebastian Ruiz-Blais, Michele Orini, Elaine Chew

**Affiliations:** ^1^School of Electronic Engineering and Computer Science, Queen Mary University of London, London, United Kingdom; ^2^Department of Clinical Science, Institute of Cardiovascular Science, University College London, London, United Kingdom; ^3^CNRS – UMR9912/STMS (IRCAM), Paris, France

**Keywords:** HRV, singing, togetherness, coherence, synchronization

## Abstract

Singing and chanting are ubiquitous across World cultures. It has been theorized that such practices are an adaptive advantage for humans because they facilitate bonding and cohesion between group members. Investigations into the effects of singing together have so far focused on the physiological effects, such as the synchronization of heart rate variability (HRV), of experienced choir singers. Here, we study whether HRV synchronizes for pairs of non-experts in different vocalizing conditions. Using time-frequency coherence (TFC) analysis, we find that HRV becomes more coupled when people make long (> 10 s) sounds synchronously compared to short sounds (< 1 s) and baseline measurements (*p* < 0.01). Furthermore, we find that, although most of the effect can be attributed to respiratory sinus arrhythmia, some HRV synchronization persists when the effect of respiration is removed: long notes show higher partial TFC than baseline and breathing (*p* < 0.05). In addition, we observe that, for most dyads, the frequency of the vocalization onsets matches that of the peaks in the TFC spectra, even though these frequencies are above the typical range of 0.04–0.4 Hz. A clear correspondence between high HRV coupling and the subjective experience of “togetherness" was not found. These results suggest that since autonomic physiological entrainment is observed for non-expert singing, it may be exploited as part of interventions in music therapy or social prescription programs for the general population.

## 1. Introduction

There is increasing interest in the effect of music on people's well-being and health. Specifically, a number of studies have shown the benefit of regular choral singing practice (Clift and Hancox, 2010; Dingle et al., 2013; Judd and Pooley, 2014; Pearce et al., 2015). Clift and Hancox (2010) identified possible factors contributing to the health and well-being benefit of choir participation, such as gaining more positive affects, focused attention, deeper breathing, social support, cognitive stimulation, and regular commitment. Dingle et al. (2013) determined three major outcomes of singing: personal (e.g., emotion regulation and spiritual experience), social (e.g., connectedness with other choir members), and functional (e.g., health benefits) outcomes. It has also been proposed that vocalizing together offers an efficient way to create bonds, which was likely an important adaptive trait for our human ancestors (Dunbar, 2017). Singing can occur in a variety of social contexts, such as amongst sport fans and within military and religious organizations. The effects of singing can be appreciated in objective health and behavioral outcomes but also in terms of the subjective qualities associated with it. Specifically, a subjective experience of togetherness is often reported in ensemble music performance and improvization (Nachmanovitch, 1990), particularly for singing (Hayward, 2014). Such experience has been described as a blurring between the self-other boundaries (Nachmanovitch, 1990), which has been linked to social bonding (Tarr et al., 2014).

Subjective experiences of togetherness have been previously studied in the context of dance (Himberg et al., 2018) and synchronized movement (Noy et al., 2015). These studies point out that interpersonal movement synchrony plays an important role in subjective experiences and aesthetic appreciation. A plausible framework through which to understand togetherness is the concept of interpersonal entrainment, which is a commonly studied phenomenon in music. Entrainment involves independent systems that become synchronized (Clayton, 2012). Four levels of interpersonal entrainment have been proposed for music (Trost et al., 2017): perceptual (the synchronization that occurs between people attending to the same stimulus), autonomic physiological [phase-locking in the activity of the autonomic nervous system (ANS)], motor (the coupling of physical actions), and social (the synchronization of social behavior). For the specific case of singing, interpersonal synchronization can occur at all the above levels: a motor (making the same vocal actions using breath and vocal chords), perceptual (listening to the same vocal sounds), autonomic physiological (the relationship between breathing and autonomic nervous system functions), and social (the communicative aspects of using the voice).

The ANS relates to emotion and behavior by means of the sympathetic and parasympathetic systems, which prepare the organism for action and regulate responses (Porges, 2001). Among relevant actions for individuals are those relating to social interaction, such as facial and vocal expressions, which are ubiquitous in singing interactions. One common way of assessing ANS activity is by analyzing the patterns of heart rate variability (HRV), which is “the degree to which the time interval between successive heart beats fluctuates” (Christou-Champi et al., 2015). HRV has a high frequency (HF) component between 0.15 and 0.4Hz, which is linked to the vagal parasympathetic activity, and a low frequency (LF) component between 0.04 and 0.15Hz, which is related to both sympathetic and parasympathetic influences (Saul, 1990). Respiration has an important effect on HRV, called respiratory sinus arrhythmia (RSA), with instantaneous heart rate increasing during inhalation and decreasing during exhalation (Song and Lehrer, 2003; Grossman and Taylor, 2007; Sin et al., 2010). Furthermore, the magnitude of the effect depends on respiration frequency, with lower frequencies showing greater RSA, with a maximum at four breaths per minute (Song and Lehrer, 2003).

It has been proposed that to understand the complexities of social interaction it is necessary to study the behavioral and physiological dynamics of various individuals (De Jaegher et al., 2010). For example, when tapping to a beat, participants adapt one to another, which is an emergent property of dyadic interactions and cannot be studied by looking at individuals separately (Konvalinka et al., 2010; Spiro and Himberg, 2012). Indeed, there is an increasing interest in studying interpersonal autonomic physiology and connecting it with behavioral and psycho-social constructs (Palumbo et al., 2017). In particular, Noy et al. (2015) studied the relationship between dyadic joint hand movements, physiological signals, and subjectively reported *togetherness* by using a mirror game inspired by theater practice (Noy et al., 2011). They found that periods of the interaction when both participants reported high togetherness where associated with increased cardiovascular activity and with high correlation between the heart rate time series of both participants (Noy et al., 2015). Their findings support the hypothesis that subjective togetherness is linked to the coupling between instantaneous heart rates of dyads, although the authors cautioned that the coupling could be a by-product of motion synchronization, for the specific task they used.

The significance of the autonomic nervous system (ANS) entrainment in group singing has been shown by Müller and Lindenberger (2011) and Vickhoff et al. (2013). Müller and Lindenberger (2011) provided the first evidence that heart rate variability (HRV) synchronizes between choir members and their conductor and that the effect is greater when singing in unison. Vickhoff et al. (2013) showed that HRV is coupled between choral singers and is dependent on musical structure, which constrains the respiration patterns. These studies suggest that HRV synchronization between choir members occurs due to RSA. However, given the link between entrainment and affective responses (Trost et al., 2017) and the socio-biological bonding responses to singing (Kreutz, 2014), it is possible that mechanisms other than RSA play a role in the HRV coupling occurring in singing interactions.

By comparing heart and respiration activity on various vocalization and breathing tasks, this study tests whether there is a mechanism beyond RSA mediating HRV coupling in dyads. HRV coupling between participants can be studied using a time-frequency coherence (TFC) analysis, which describes the amount of coupling between two signals over different frequencies (Orini et al., 2012b, 2017a). Furthermore, partial time-frequency coherence (pTFC) provides a means to study the coupling between two signals after removing the effects of a third signal (Orini et al., 2012a,b; Widjaja et al., 2013). We use pTFC to study the coupling between the HRV of dyads beyond the effects of respiration. We expect that, by removing the effect of respiration, there will be no differences in pTFC between baseline and breathing conditions. We propose that some differences might remain between breathing and vocalization conditions, due to influences beyond RSA. Furthermore, this study explores whether HRV synchronization relates to the subjective experience of togetherness, by using continuous subjective ratings of togetherness (Noy et al., 2015). The differences between making short and long vocalizations and making them in-sync or out-of-sync are also explored. We thus attempt to provide insight into the physiological effects of specific characteristics of vocalization, i.e., length and degree of synchrony between people, which shape more complex forms of vocalization such as choir singing. While choir singing involves more elements than this specific case of dyadic vocalization, this experimental design allows the study to isolate some aspects of singing (e.g., length and synchrony) while preserving the singing experience to some extent (e.g., by giving participants some freedom in the choice of their notes). Finally, HRV synchronization has not been demonstrated for people without singing experience. We aim to reproduce this phenomenon in a non-expert population in order to contribute to research on the use of singing in music therapy contexts.

## 2. Methodology

The study received ethical approval by the Research Ethics Committee of Queen Mary University of London.

### 2.1. Participants

Twenty participants (10 male and 10 female) aged 20–43 were paired in 10 dyads for a vocal interaction experiment. We recruited participants who identified themselves as non-expert singers to extend previous results to people without regular choir or singing practice. Participants were given an information sheet and provided written informed consent. Among the group of 20 participants, one dyad dropped out of the analysis because the participants laughed intermittently, hence affecting the physiological measurements. In addition, continuous subjective ratings from two participants were lost due to technical issues. We thus used data from 18 participants (nine dyads) for the physiological analyses and data from 16 participants for the subjective ratings analyses.

### 2.2. Procedure

Each dyad was guided through the following phases: briefing, physiological sensors set-up, a warm-up phase, four tasks of vocal interaction, a continuous subjective rating phase, a questionnaire, and an interview. We performed baseline recordings for 1 min before and 1 min after the interactive tasks. The whole procedure lasted about 70 min and participants were compensated with £10 for their time. During the briefing, participants completed the consent forms, and the experiment was explained.

For both the warm-up and the four interactive tasks, participants sat on chairs about 1 m apart and both facing a common central point. This configuration was chosen in part due to the size constraints of the room and to encourage participants to use their peripheral vision for the interaction while not facing each other directly. Participants could thus choose whether or not to make eye contact when interacting. For the subjective ratings, questionnaire, and interviews, participants were each in a different room.

The warm-up phase was designed to give participants awareness of their own voice by exploring different sound parameters, such as pitch, intensity, and duration. Participants were guided through the warm-up one at the time. The experimenter prompted the participants with vocal sounds that they had to imitate immediately after hearing the sounds. The warmup started with a short, mid-range tone, progressing gradually to higher pitches followed by lower pitches. Next, high and low intensities were presented following a similar pattern. Finally, the participants heard and mimicked two long notes; this was to make sure the participants could control their breathing effectively. In all the vocalized tasks participants were encouraged to explore different pitches and intensities freely to give a greater sense of agency, showing in the different choices made by different dyads. Furthermore, while participants were asked to make short notes of about 0.5 s and long notes as long as their breath, they had some freedom in their choices, both to provide a sense of agency and simplify the task. Each task lasted between 90 and 120 s. A short explanation was given before each task, and participants were asked to return to normal breathing at the end of the task.

In the first task (Br), participants were asked to synchronize their breathing without previously agreeing on any strategy. The second task (SNsync) consisted of synchronizing short duration notes. Participants were asked to achieve synchronization without explicitly agreeing to any kind of strategy. In the third task (LN), participants were asked to make synchronized notes of long duration, paying attention to both the beginnings and ends of the notes. Participants were asked to vocalize pitched sounds for the duration of the respiration and to prioritize synchronization over note length, meaning that if a participant would run out of air the other would have to stop as well. In the fourth task (SNasync), participants produced out-of-phase short notes with the constraint of not vocalizing at the same time, but they were otherwise free to choose the timings of their vocalizations.

### 2.3. Data Recording

#### 2.3.1. Audio and Video

Audio was recorded using a ZOOM H4 recorder at a standard 44,100 Hz sampling rate, and video was recorded with the in-built camera of a MacBook Air using the Photobooth application. A frame where both participants were visible was chosen. Both audio and video recordings were started a few seconds after the beginning of the breathing task and were stopped a few seconds after the end of the asynchronous notes task. Audio and video signals were synchronized using MATLAB's “finddelay” function with a maximum delay of 20 s.

#### 2.3.2. Togetherness Continuous Subjective Ratings

**Figure 1:**
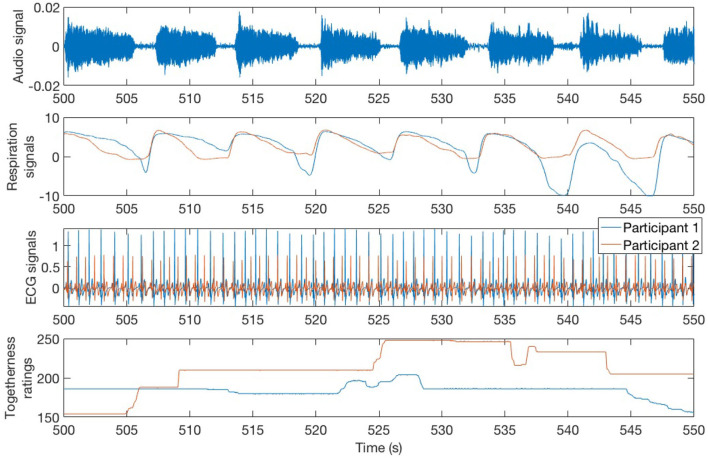
A sample of the synchronized audio, respiration, and ECG signals and togetherness ratings for both participants in dyad 1 during the synchronized Long Notes condition.

Participants were asked to report the degree of togetherness they experienced with their partner throughout the four interactive tasks, as in previous studies (Noy et al., 2015). Togetherness was defined to the participants as “the extent to which you feel close or connected to your partner." Immediately after the experimental tasks, participants were taken to separate rooms and shown the video recording of the interaction. They were asked to report how much togetherness they experienced during the tasks, using continuous subjective ratings. A rating dial and a visual interface were provided, and they recorded numeric values between 0 and 255 and then normalized to the 0–1 range during the analysis. Participants were instructed to turn the dial to the left side to register low togetherness values and to the right side to register high values. The interface provided visual feedback on the level of togetherness that was reported. The interface was created using Arduino hardware and Processing software. The software included timestamps to allow synchronization between the video and physiological data. See Figure 1 for an example of continuous togetherness ratings for one of the dyads.

#### 2.3.3. Physiological Data

Physiological data was continuously recorded for each participant, during the four interactive tasks and baselines, using the BIOPAC MP150 system and software AcqKnowledge. ECG was recorded using three leads (BN-EL30-LEAD3) and a standard configuration with the white active electrode on the right upper chest, the black ground electrode on the left upper chest, and a red active electrode on the left lower chest. Respiration depth was recorded using the BIONOMADIX respiration belt. Signals from both participants were simultaneously recorded using a sampling rate of 1,000Hz. Timestamps were used to synchronize the physiological data with audio, video, and continuous subjective ratings. See Figure 1 for an example of the respiration and ECG signals.

We recorded baseline physiological data for 1 min before and after the block of four tasks, during which the participants were asked to breathe normally and relax. For each measure, we computed the average between the initial and final baselines to get single baseline measures (Bs). We also recorded about 20–25 s of data between the tasks allowing the physiological signals to return to baseline.

### 2.4. Analysis

#### 2.4.1. Physiological Measures

Respiration signals were re-sampled at 4 Hz and a band-pass filter within [0.04–1] Hz was applied to reduce noise introduced by the equipment. For each participant, the RR intervals were obtained from the ECG data using a semi-automated MATLAB GUI as in previous studies (Orini et al., 2017b), which allows for revision and manual correction. Ectopic beats and artifacts were rare, and they were interpolated when present. The RR interval series was re-sampled at 4 Hz and the heart rate variability signal was obtained by high-pass filtering these series with a cut-off frequency of 0.03 Hz.

We computed the mean heart rate (HR) and the Root Mean Square of Successive Differences (RMSSD) between adjacent RR intervals for each participant and each condition (baseline, breathing in synchrony, short notes in synchrony, long notes in synchrony, and asynchronous short notes). HR is a measure of cardiovascular activity, and RMSSD is as common measure of HRV revealing how much the RR intervals fluctuate (Christou-Champi et al., 2015).

We applied the same methodology used in Orini et al. (2012b) to obtain the time-frequency coherence between two signals, which gives the correlation between two signals at different frequencies. The time-frequency coherence is defined as follows:

(1) $$\begin{array}{r} \gamma_{xy}\left(t,f\right)=\frac{\left|S_{xy}\left(t,f\right)\right|}{\sqrt{S_{xx}\left(t,f\right)S_{yy}\left(t,f\right)}}, \end{array}$$

where *S*_*xy*_(*t, f*) is the cross-power spectral density of signals *x*(*t*) and *y*(*t*), which in this study represent HRV or respiration signals from each one of the participants, and is computed over time:

(2) $$\begin{array}{r} S_{xy}\left(t,f\right)=F\left\{E\left[x\left(t+\frac{\tau }{2}\right)y^{*}\left(t-\frac{\tau }{2}\right)\right]\right\}, \end{array}$$

and *F*{·} and *E*[·] are the Fourier transform and the expectation operators, respectively (Orini et al., 2012b).

Although the frequencies of interest to analyze HRV are typically in the range 0.03–0.4 Hz, we were also interested in potential effects of short and fast vocalizations (up to one note per second) and performed the analysis in the range of 0.03–1 Hz.

In order to test the effect of respiration on HRV coupling, we computed the arithmetic mean of the TFC in the respiratory band, using the average of the respiratory frequency of both participants. The respiration frequency was determined for each participant as the peak frequency of the time-frequency spectrum of respiration. The respiratory band was defined by a window around the frequency of the respiration signal, with a width twice the frequency resolution of the time-frequency coherence analysis (0.078 Hz), as in previous studies (Orini et al., 2012c). The band was restricted to the [0.04–1] Hz range. An arithmetic mean was then obtained over time for each condition separately. This provided a coherence index for each condition for each dyad.

We additionally computed a partial time-frequency coherence (pTFC), which assesses the coupling of two signals after removing the effects of a third signal (Orini et al., 2012a; Widjaja et al., 2013). In this case, it was used to determine whether there was coupling beyond the effects of respiration. The pTFC function is defined as follows:

(3) $$\begin{array}{r} \gamma_{xy/z}\left(t,f\right)=\frac{\left|S_{xy/z}\left(t,f\right)\right|}{\sqrt{S_{xx/z}\left(t,f\right)S_{yy/z}\left(t,f\right)}}, \end{array}$$

and *S*_*xy*/*z*_(*t, f*) is the partial cross-power spectral density, obtained as follows:

(4) $$\begin{array}{r} S_{xy/z}\left(t,f\right)=S_{xy}\left(t,f\right)-\frac{S_{xz}\left(t,f\right)S_{zy}\left(t,f\right)}{S_{zz}\left(t,f\right)}. \end{array}$$

For our purposes, the third signal *z*(*t*) was the respiration data from one of the participants. Because the respiration signal from either participant could be used to obtain the pTFC, we computed a pTFC using respiration signals from each participant and then averaged the two pTFCs. We averaged the pTFC over frequencies, although in this case we used the full range (0.03 − 1 Hz) rather than the respiratory band. Then, as for the TFC, we averaged the results over time to obtain one coherence index per condition.

#### 2.4.2. Statistical Analyses

The measures we used in the statistical analyses were HR, RMSSD of HRV, and average togetherness ratings for individuals and TFC and pTFC for dyads. The Kolgomorov-Smirnov test for normality showed that the distributions were not normal. We thus used the non-parametric Wilcoxon sign rank tests for all analyses, allowing for paired comparisons. We used the Holm-Bonferroni method for multiple comparison correction (Holm, 1979). This consists of ordering the p-values from lowest to highest (*p*_*k*_, with *k* = 1:*M*, where *M* is the number of comparisons), and then rejecting the null hypothesis for comparisons for which *p*_*k*_ < 0.05/(*M* − *k* + 1). Once a null hypothesis is rejected the procedure is stopped. For HR, RMSSD, TFC of respiration, and TFC of HRV we were interested in seven comparisons:

- between baseline (Bs) and each condition (Br, SNsync, LN, and SNasync) to test each condition relative to the control;
- between Br and LN to test the effect of voice;
- between SNsync and LN to test the effect of the length of the vocalizations; and
- between SNsync and SNasync to test the synchrony of the vocalizations.

For pTFC, we were only interested in the effect of voice, and performed only three comparisons: LN with Br, LN with Bs, and Br and Bs. The latter allowed us to ensure that there was no coupling for the breathing condition. For the subjective ratings of togetherness, we performed three comparisons: Br and LN, SNsync and LN, and SNsync and SNasync.

### 2.5. Interviews and Questionnaire

Interviews were conducted to determine the strategies used by the participants to accomplish the tasks and to better understand the way people understand the concept of togetherness. During the interviews, participants were asked to report the aspects that made the tasks engaging, the differences between the tasks regarding their experience of pleasure, engagement, and connection with the other, and the aspects of the interaction contributing to the experience of togetherness. A questionnaire was also used to collect some information such as how challenging the task was for the participants (on a scale from 1 to 10) and to what extent they knew each other.

## 3. Results

Table 1 shows the mean and standard deviation for HR, RMSSD, respiration frequency, TFC of respiration, and HRV signals averaged in the respiration band, partial TFC of HRV, and subjective togetherness values.

**Table 1:** Means and standard deviations of heart rate (bpm), RMSSD of heart rate variability (ms), average respiratory frequency (Hz), time-frequency coherence of respiration signals averaged in the respiration band, time-frequency coherence of HRV averaged in the respiratory band, partial TFC average, and subjective togetherness for each experimental condition.

	**HR**	**RMSSD**	**Resp. freq**.	**Resp TFC**	**HRV TFC**	**pTFC**	**Together**.
Baseline	76.4(7.9)	7.2(3.1)	0.63(0.27)	0.58(0.21)	0.32(0.10)	0.12(0.03)	N/A
Breathing	75.8(9.4)	10.6(4.0)	0.25(0.08)	0.91(0.05)	0.86(0.06)	0.13(0.04)	0.52(0.24)
SNsync	76.4(8.9)	8.8(3.1)	0.36(0.20)	0.53(0.18)	0.52(0.13)	0.12(0.04)	0.66(0.14)
LN	74.8(7.3)	14.5(4.7)	0.11(0.04)	0.88(0.06)	0.87(0.09)	0.21(0.07)	0.70(0.14)
SNasync	78.4(9.0)	8.8(2.8)	0.17(0.08)	0.50(0.13)	0.53(0.07)	0.12(0.03)	0.67(0.20)

*Togetherness values are normalized to the 0–1 range*.

### 3.1. Heart Rate and RMSSD of HRV

**Figure 2:**
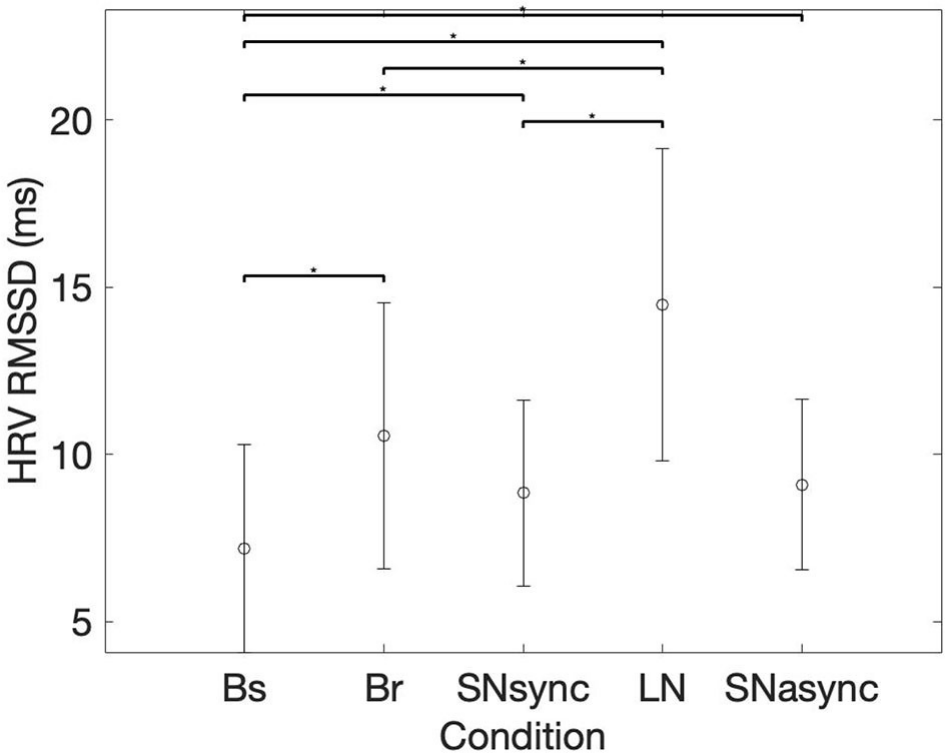
Mean (circles) and SD (bars) for the RMSSD of Heart Rate Variability. *Represents statistically significant differences, using Holm-Bonferroni correction.

Results for heart rate and RMSSD of HRV are summarized in Table 2 and Figure 2. There was no difference in the averaged HR between conditions (Br, SNsync, LN, and SNasync). We found that RMSSD was greater for Br (*p* = 0.0016), SNsync (*p* = 0.011), LN (*p* = 0.0002), and SNasync (*p* = 0.0074) compared to Baseline, for LN compared to Br (*p* = 0.0006), and for LN compared to SNsync (*p =* 0.0002).

**Table 2:** Comparisons between conditions for heart rate, RMSSD of heart rate variability, time-frequency coherence of respiration signals averaged in the respiration band, time-frequency coherence of HRV averaged in the respiratory band, partial TFC average and subjective togetherness values.

	**HR**	**RMSSD**	**Resp TFC**	**HRV TFC**	**pTFC**	**Togeth**.
**Comparison**	***p*-value**	***p*-value**	***p*-value**	***p*-value**	***p*-value**	***p*-value**
Bs and Br	0.9133	0.0016^*^	0.0078^*^	0.0039^*^	0.5703	N/A
Bs and SNsync	0.8107	0.0108^*^	0.4961	0.0117^*^	N/A	N/A
Bs and LN	0.1701	0.0002^*^	0.0078^*^	0.0039^*^	0.0117^*^	N/A
Bs and SNasync	0.0778	0.0074^*^	0.3594	0.0039^*^	N/A	N/A
Br and LN	0.4204	0.0006^*^	0.1641	0.4258	0.0078^*^	0.0174
LN and SNsync	0.2668	0.0002^*^	0.0039^*^	0.0039^*^	N/A	0.1961
SNsync and SNasync	0.0778	0.8107	0.3008	1	N/A	0.3794
Number of comparisons	**7**	**7**	**7**	**7**	**3**	**3**

* *Indicates statistical significance using Holm-Bonferroni correction*.

### 3.2. TFC of Respiration

The results of the TFC between respiration signals are shown in Table 2. The TFC of respiration for Br and LN was significantly higher than for Bs (*p* = 0.0078) and for LN than SNsync (*p* = 0.0039). Respiration signals were not more synchronized for SNsync or SNasync compared to Bs.

### 3.3. HRV Coherence in Respiratory Band

**Figure 3:**
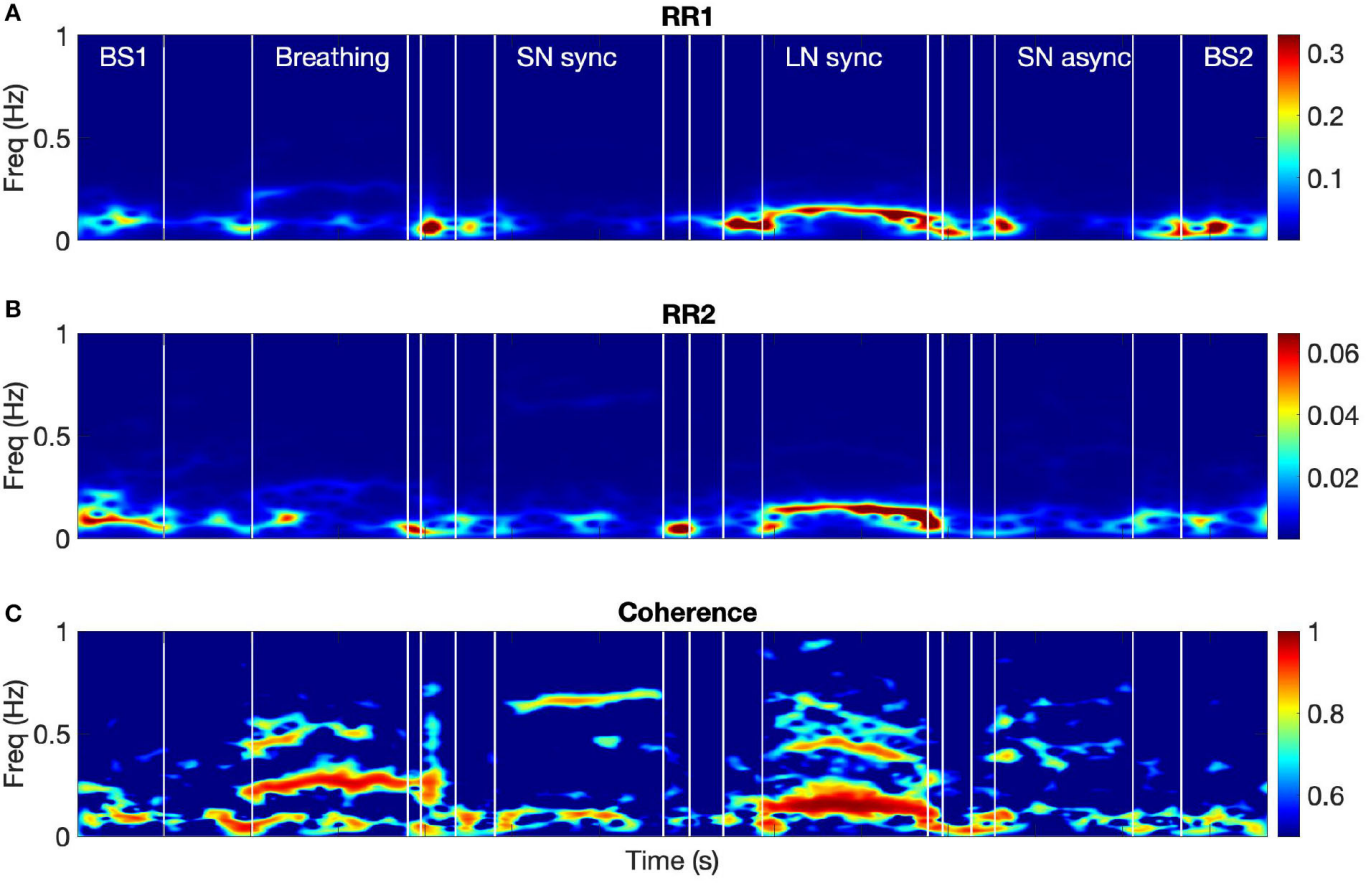
The spectra of the RR intervals for both participants from dyad 1 **(A,B)** and their coherence spectrum **(C)**. BS1 and BS2 refer to the 60-s baselines before and after the tasks, respectively. Breathing refers to the breathing condition and LN to the long notes condition. SNsync and SNasync refer to the conditions with synchronous and asynchronous short notes, respectively.

**Figure 4:**
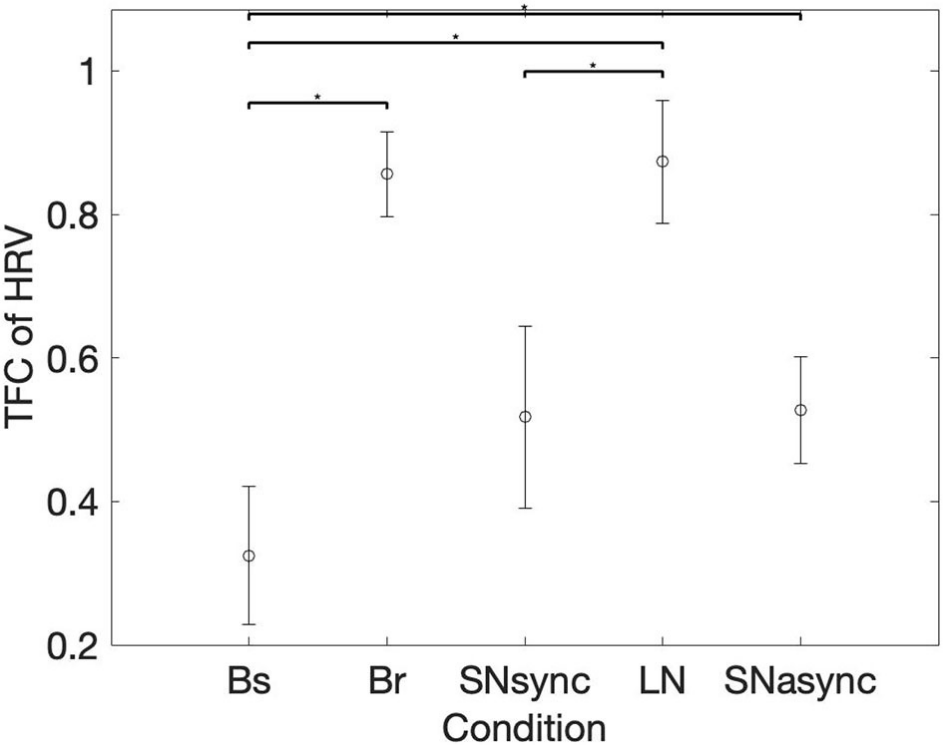
Mean and SD for the TFC average on respiration band. *Represents statistically significant differences using Holm-Bonferroni correction.

Figure 3 shows the time-frequency coherence between HRV for dyad 1. It can be appreciated that there is an increase in coherence in Br and LN conditions for a range of frequencies, with peaks around 0.3 and 0.1 Hz and harmonic components at multiple frequencies. The average coherence in the respiratory band was greater for Br, LN, and SNasync than Bs (*p* = 0.0039), for SNsync than Bs (*p* = 0.0117), and for LN than SNsync (*p* = 0.0039). All results are summarized in Table 2 and Figure 4.

**Figure 5:**
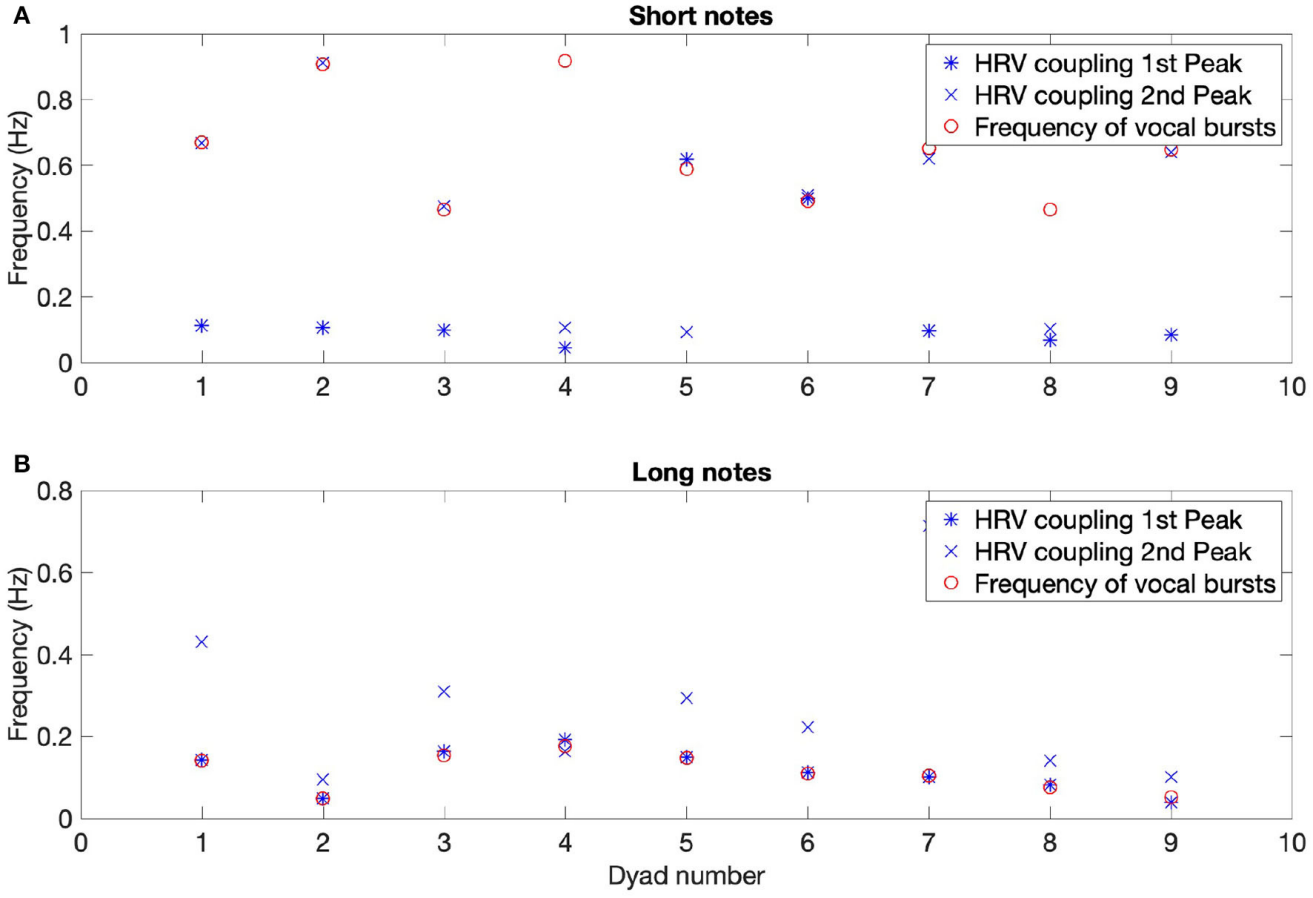
Mean frequency of HRV coherence peaks and of vocal bursts for each dyad, for synchronous short **(A)** and long notes **(B)**. The correspondence between the frequency of vocal bursts and the first peak in the HRV coherence is striking for long vocalizations **(B)**. For short vocalizations, there is a correspondence between the frequency of onsets and one of the first two peaks in the HRV coherence for seven out of nine dyads **(A)**.

A stable component at very high frequency (between 0.4 and 0.9 Hz) was present in the time-frequency coherence between HRV for most dyads. To investigate this in more detail we examined the relationship between the peak frequencies in the time-frequency coherence between HRV and the frequency of the vocal bursts (the inverse of the time between the beginnings of successive bursts). Moving average was applied to the audio signals to determine the onsets of the vocal bursts and thus their frequency. In the SNsync condition, participants produced notes every 1.6 s on average (range of 1–2.5 s), corresponding to 0.64 Hz. For seven out of nine dyads, the average frequency of vocal bursts matched either the first or second peak in the corresponding HRV coherence spectra averaged over time for the SN and LN conditions (see Figure 5). This effect is even clearer for LN, with 9 out of 9 dyads showing a correspondence between the first peak in the HRV coupling and the frequency between bursts. Because the vocal pattern imposes a respiratory rhythm, we conclude that for SNsync and LN there is an effect of breathing on HRV.

There was no significant difference between the TFC of HRV of LN and Br. Additionally, when analyzing the time intervals between successive exhalations using the respiration signals for both conditions, we found that, on average, the period of respiration for the Br condition was of 2.8 s (range of 3.5–5.5 s) vs. an average of 9.1 s (range of 5–22 s) for the LN condition. Participants were thus having longer breathing cycles for LN than for Br, which we discuss in section 4.

### 3.4. HRV Partial Coherence

**Figure 6:**
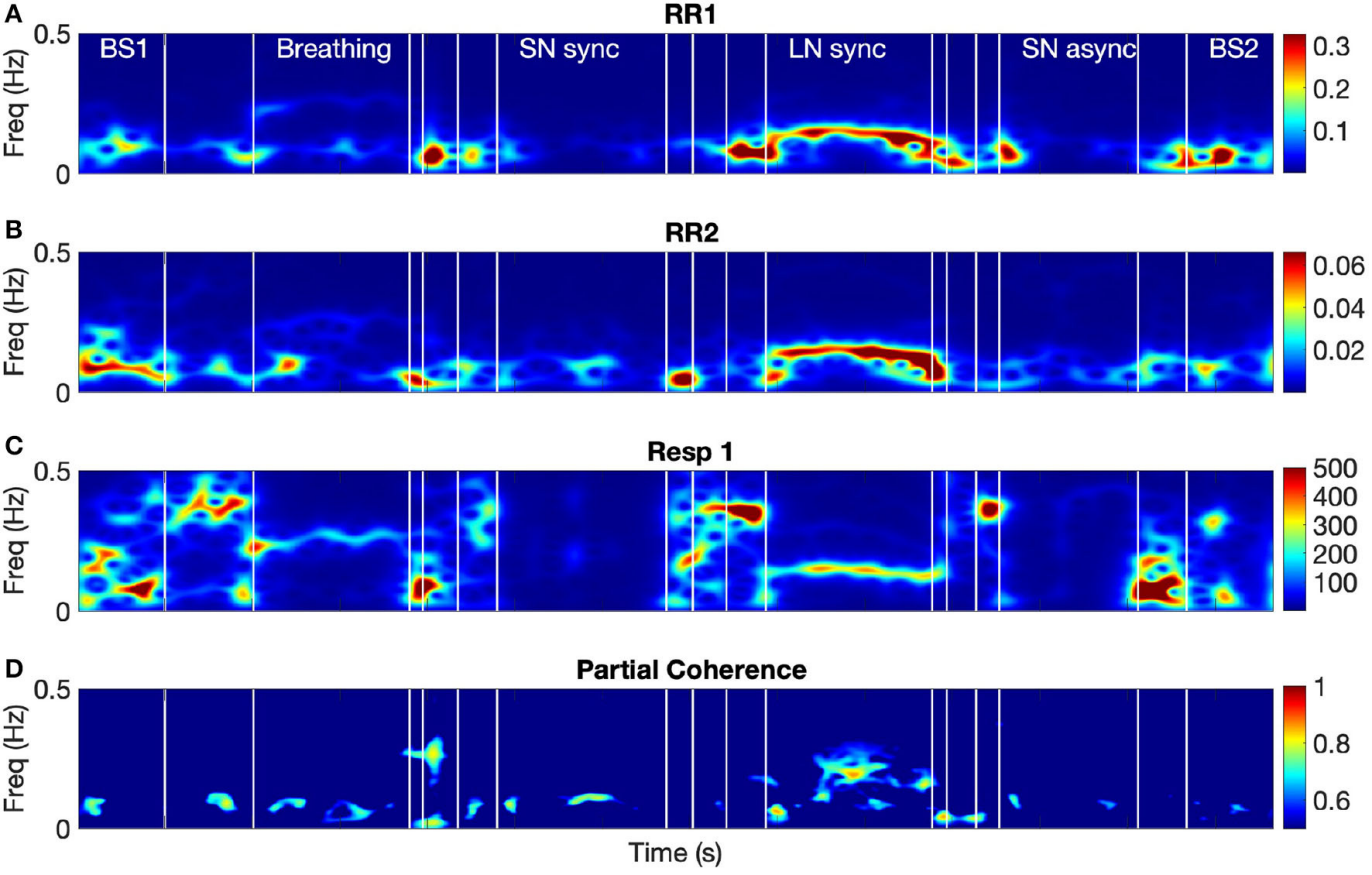
The spectra of the RR intervals for both participants from dyad 1 **(A,B)**, the respiration signal from participant 1 **(C)**, and their partial time-frequency coherence **(D)**. Name of the conditions is the same as in Figure 3.

**Figure 7:**
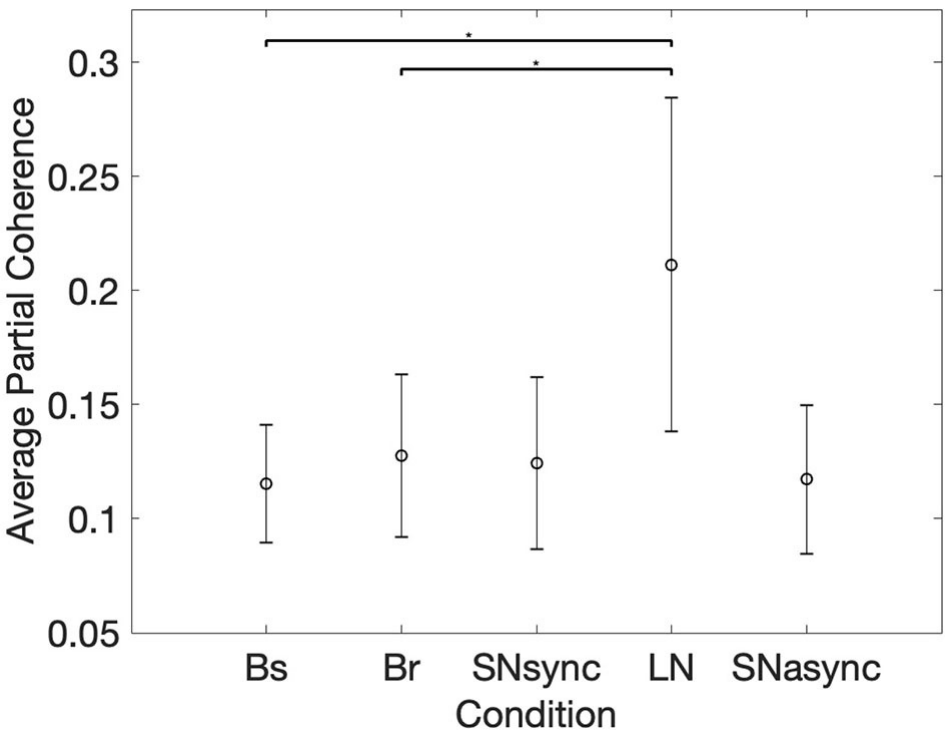
Mean and standard deviation of the average of time-frequency coherence after removing the respiration component. *Represents statistically significant differences using Holm-Bonferroni correction.

In order to determine changes in HRV coherence not related to RSA, we computed the pTFC, which removes the respiratory component from the TFC of the dyad's HRVs (see Figure 6). We hypothesized that a significant difference between the long notes and breathing conditions after removing the respiration component would indicate the presence of another mechanism beyond RSA. Results are summarized in Table 2 and Figure 7. Partial TFC was higher during LN than Bs (*p* = 0.0117) and Br (*p* = 0.0078), suggesting that for long notes coupling between HRV in the two participants occurred beyond the effect of breathing. We also found no differences in pTFC during Br vs. Bs conditions, which was expected since these conditions only differ in the breathing pattern and partial coherence removes the effect of breathing. For LN, the average of the TFC on the 0–1 Hz range decreased from 0.87 to 0.21 when removing the effects of respiration (see Table 1), suggesting the effect of RSA predominantly mediates the HRV coupling.

### 3.5. Togetherness

We compared the mean values of togetherness' subjective ratings between Br and LN, SNsync and LN, and SNsync and SNasync. We found no that subjective togetherness was only greater for LN compared to Br (*p* = 0.017), although this remains a trend as results were not significant after correcting for multiple comparisons. The other comparisons were not statistically significant. Participants generally agreed regarding the preferred conditions, indicated by a higher mean in the reported togetherness. LN had the highest mean for nine participants, SNasync was preferred by five participants, SNsync was preferred by two, and Br was preferred by none. The differences between LN and Br suggest that the presence of voice has an important effect on the subjective experience of togetherness. The lack of a statistically significant result might be due both to sample size and the noisy nature of these subjective reports. Interestingly, the SNasync condition was the second preferred condition, suggesting factors other than synchrony are relevant for participants when rating togetherness.

Three kinds of togetherness experiences emerged from the interviews. First, some participants referred to the experience with words such as “existential” or “meditative” and reported it was an “intimate experience” allowing to have a joint expression with someone else. For instance, some people reported having felt more connected than they would by means of conversation. Second, particularly with regards to the asynchronous condition, some participants were engaged by the fact that the interaction was “playful,” and that they could come up with ideas more freely than in the synchronous ones. The possibility of responding to each other asynchronously allowed for a call and response game and hence appraised as more interactive. Third, participants found that having a common goal and pursuing it as a team contributed to their experience of togetherness. Some participants reported that they experienced less togetherness in more chaotic parts of the interaction, while more “harmonic” parts gave rise to more togetherness. We speculate that more chaotic interactions could be interpreted as an absence of a common goal by some participants. The previous themes indicate that the construct of togetherness can be divided into at least three different components, which we introduce here as the *existential, playful*, and *common-goal* togetherness.

We were also interested in exploring how the self-reported challenge level of the tasks could relate to the experienced togetherness. We found no significant differences in how the participants rated the challenge level of each task and the correlation between the subjectively reported challenge level and the average togetherness was very weak (*r =* 0.19).

## 4. Discussion

This study shows that synchronization of respiration mediates HRV coupling when non-experts vocalize together, expanding upon previous results (Müller and Lindenberger, 2011; Vickhoff et al., 2013). By comparing the strength of the coupling before and after removing the respiration signal, we conclude that RSA accounts for a significant part of the effect. The finding that HRV coupling was higher after analytically removing the respiration component from the TFC for synchronized long notes but not for synchronized breathing suggests that a mechanism other than RSA also contributes to HRV coupling when vocalizing together. The main difference between LN and Br is the presence of voice, suggesting that either synchronization of vocal muscular action or perception of voice might mediate HRV coupling. Since the vagus nerve links the vocal chords, facial expressions, and heart rate (Porges, 2001), it may be possible that the voice affects HRV by means of the ANS.

The analysis of the audio recordings shows that the frequency of vocal bursts and HRV peaks matched for both synchronized, short, and long note conditions. Differences between synchronized short and long vocalizations can be due to various reasons, such as different physiological mechanisms operating at different frequencies. For the specific tasks that were used, when making long notes, people synchronized both the beginnings and ends of the vocalizations; for short notes, however, people inhaled at different times. In addition, very short notes, made every 1 or 2 s, are likely to have a frequency that is close to the heart rate, and hence are less likely to appear in the spectral analysis due to the heart rate being the natural sampling frequency. Our results show that although frequencies above 0.4 Hz are typically not considered in the HRV analyses, some coupling persists even at higher frequencies, and the respiratory spectral band should be adjusted to respiration (Orini et al., 2012c). An implication of these results is the possibility to make vocal interventions targeting HRV coupling at specific frequencies.

The time-frequency coherence of respiration and of HRV do not match for SNsync and SNasync (see Table 2), suggesting respiration synchrony does not mediate the observed HRV entrainment for short notes. However, in the context of the tasks that were used in this study, the breathing patterns were not controlled and hence the quality of the respiration signals might have been different for short and long notes. While for LN and Br conditions, participants made deeper and more synchronized breaths that fluctuated between two values, for the short notes conditions (SNsync and SNasync), they had freedom to inhale between each pair of vocalizations or to take longer breaths, inhaling only occasionally. Furthermore, the fact that TFC of HRVs was higher during SNasync than during Bs may indicate that participants' vocalizations were coupled, even if they were explicitly asked to perform their notes at different times. We observed that for most dyads and in the SNasync condition, participants timed their short notes in response to their partners (as in the call and response dynamic noted earlier), hence producing some degree of synchrony in the TFC analyses, which yields high values for phase-delayed signals.

Musical entrainment usually refers to the entrainment to a musical beat, which is only possible for frequencies above 0.5 Hz, with a period between beats lower than 2 s (Repp and Doggett, 2007). In our study, only the synchronized short notes condition allowed for such entrainment. Because we found a stronger HRV coupling for vocalizations of longer duration, we conclude that HRV entrainment is primarily due to RSA and is independent of beat entrainment. This is consistent with the four levels of entrainment proposed by Trost et al. (2017) and stresses that aspects other than those related to musical tempo entrain during music interaction and might play a role in affective states. This makes a case for studying music with weak or no sense of beat, as is found in many segments of traditional music and some types of contemporary music, such as drone, ambient, and soundscape genres.

HRV is affected by emotional arousal and valence (Orini et al., 2010, 2019) and is considered a “biomarker of successful emotional regulation,” which is the capacity of an individual to maintain positive emotions despite unfavorable contexts (Christou-Champi et al., 2015). Individuals regulate their emotions using slow paced breathing (Song and Lehrer, 2003) presumably by imposing a rhythm on the heart activity patterns, affecting the rest of the body and the brain. The heart-brain connection is being increasingly studied (Dunn et al., 2010; Mather and Thayer, 2018) and RSA has been effectively exploited to affect psychological states (Lehrer and Gevirtz, 2014). One of the possible implications of HRV entrainment between people is a potential role in bonding, by simultaneously affecting the psycho-physiological state (Bernardi et al., 2017) or by facilitating coordination by means of synchronizing inner rhythms (Vickhoff et al., 2013). These are yet to be supported by research.

Contrary to our initial hypothesis, we did not find a strong correlation/interaction between dyadic HRV coupling and a subjective experience of togetherness. In fact, the subjective experience of togetherness is a complex construct and unlikely to be reducible to a physiological marker. We speculate that at least three factors contribute to the subjective experience of togetherness: having a common-goal, playfulness, and existential togetherness. The common-goal factor likely operates at a more abstract level, involving cognitive appraisals of joint success in the task. We assume that this component is not related to autonomic physiological synchrony, because all participants can simultaneously have different appraisals of the same situation. The playful aspect seems to be closely linked to language in the sense that it relates to a call and response interaction. It was mostly reported with regards to the asynchronous condition, where HRV coupling was not significant. The playfulness component is therefore also unlikely to be related to autonomic physiological synchronization. Lastly, the existential aspect of togetherness involves a sense of sameness, which may arise when people are doing the same action [“we-agency”, as in Vickhoff et al. (2013)]. This is associated with “oneness” and “spiritual” experiences, typical of many singing contexts (Dingle et al., 2013). We speculate that if HRV coupling is related to a togetherness experience, the existential component of togetherness would be the most relevant. Further research is required to establish whether more specific subjective reports of existential togetherness consistently correlate with autonomic physiological synchrony.

## 5. Conclusion

This study shows that HRV of non-expert singing together shows a higher level of coupling than during baseline. We found that making synchronous long vocalizations produced greater coupling in the respiration band of the heart rate variability coherence compared to making short vocalizations. In addition, for synchronized long vocalizations but not for synchronized breathing, HRV coupling was greater than baseline after removing the effect of respiration. These results suggest that while HRV coupling was mainly driven by a synchronization of the respiratory activity, joint vocalization also contributes to HRV coupling beyond the effect of respiration.

Subjectively experienced togetherness did not show correlations with physiological synchrony, likely due to the complexity of the togetherness construct. Detailed interviews identified three main components to subjective togetherness, which we introduce here as the *existential, playful*, and *common-goal* togetherness. Future research is needed to assess the interaction between these components and autonomic physiological synchrony and the potential benefit of interventions resulting in HRV entrainment between people.

## Data Availability Statement

The raw data supporting the conclusions of this article will be made available by the authors, without undue reservation.

## Ethics Statement

The studies involving human participants were reviewed and approved by Queen Mary Ethics of Research Committee. The participants provided their written informed consent to participate in this study.

## Author Contributions

SR-B, EC, and MO contributed conception and design of the study. SR-B performed the statistical analysis and wrote the manuscript. MO provided the analysis tools. All authors contributed to discussion and manuscript revision and read and approved the submitted version.

## Conflict of Interest

The authors declare that the research was conducted in the absence of any commercial or financial relationships that could be construed as a potential conflict of interest.
